# Supplementary material for: Association of MDM2 rs2279744 and rs937282 polymorphisms with breast cancer susceptibility and circulating MDM2 and p53 levels in Bangladeshi women: A case-control study
Source: PLoS One. 2026 Sep 1;21(9):e0350622. doi: 10.1371/journal.pone.0350622 (PMC13533348; doi:10.1371/journal.pone.0350622)
Supplement: Supplementary file 1 [file pone.0350622.s001.docx]

**S1 Table: Demographic characteristics of BC cases and HC**

| **Variable** | **Cases (n=112)** | **Controls (n=124)** | **p-value*** |
| --- | --- | --- | --- |
| **Age (years)** | 46.9 ± 12.5 | 47.8 ± 13.1 | 0.58 |
| **BMI (kg/m²)** | 25.7 ± 4.9 | 25.2 ± 5.1 | 0.32 |
| **Number of Children** | 2.13 ± 0.91 | 2.09 ± 0.87 | 0.45 |
| **Occupation** |  |  | 0.46 |
| Housewife | 38 (33.9%) | 44 (35.5%) |  |
| Working Women | 68 (60.7%) | 73 (58.9%) |  |
| Student | 6 (5.4%) | 7 (5.6%) |  |
| **Level of Education** |  |  | 0.71 |
| Illiterate | 60 (53.57%) | 60 (48.38%) |  |
| <10 years | 40 (35.71%) | 48 (38.71%) |  |
| ≥10 years | 12 (10.71%) | 16 (12.9%) |  |
| **Socio-economic Status** |  |  | 0.29 |
| Lower Class | 59 (52.68) | 74 (59.68%) |  |
| Middle Class | 45 (40.19%) | 38 (30.66%) |  |
| Higher Class | 8 (7.14%) | 12 (9.68%) |  |
| **Residential Status** |  |  | 1.0 |
| Rural | 76 (67.86%) | 85 (68.55%) |  |
| Urban | 36 (32.14%) | 39 (31.45%) |  |
| **Menopausal Status** |  |  | 0.881 |
| Premenopausal | 49 (43.75%) | 52 (41.94%) |  |
| Postmenopausal | 63 (56.25%) | 72 (58.06%) |  |

- Data are shown as mean ± SD (median) for continuous variables and % (n) for categorical variables.
- *p-values: t-test for continuous variables (age, BMI, number of children); Chi-square test for occupation, level of education, socio-economic status, residential status and menopausal status.
