# Supplementary material for: Association of MDM2 rs2279744 and rs937282 polymorphisms with breast cancer susceptibility and circulating MDM2 and p53 levels in Bangladeshi women: A case-control study
Source: PLoS One. 2026 Sep 1;21(9):e0350622. doi: 10.1371/journal.pone.0350622 (PMC13533348; doi:10.1371/journal.pone.0350622)
Supplement: Supplementary file 2 [file pone.0350622.s002.docx]

**S2 Table: Histopathological data of BC Cases**

| **Variables** | **Cases (n=112)** |
| --- | --- |
| **Breast Lump** | 112 (100%) |
| **Nipple Discharge** | 21 (18.75%) |
| **Retraction of Nipple** | 16 (14.29%) |
| **Deviation of Nipple** | 10 (8.92%) |
| **Weight Loss** | 45 (40.19%) |
| **Peau d’orange** | 44 (39.29%) |
| **Ulceration of Overlying Skin and/or Nipple Areola** | 11 (9.82%) |
| **Histology** |  |
| Ductal ca in situ | 16 (14.29%) |
| Invasive Lobular | 12 (10.71%) |
| Invasive Ductal | 84 (75%) |
| **Clinical Stages** |  |
| Grade I | 52 (46.43%) |
| Grade II | 42 (37.5%) |
| Grade III | 18 (16.07%) |
| **Hormone Receptor** |  |
| *ER+* | 73 (65.18%) |
| *PR*+ | 80 (71.43%) |
| *HER2+* | 32 (28.57%) |
| **Tumor Size** |  |
| <2 cm | 6 (5.36%) |
| 2.1 – 5.0 cm | 62 (55.36%) |
| >5.0 cm | 44 (39.29%) |
| **Lymph Node** |  |
| Positive | 30 (26.79%) |
| Negative | 82 (73.21%) |
